# Dysbiosis by neutralizing commensal mediated inhibition of pathobionts

**DOI:** 10.1038/srep38179

**Published:** 2016-11-29

**Authors:** Esteban Rodriguez Herrero, Vera Slomka, Nico Boon, Kristel Bernaerts, Emma Hernandez-Sanabria, Marc Quirynen, Wim Teughels

**Affiliations:** 1 Department of Oral Health Sciences, KU Leuven, Kapucijnenvoer 33, 3000 Leuven, Belgium; 2 Center for Microbial Ecology and Technology (CMET), Ghent University, Coupure links 653, 9000 Gent, Belgium; 3 Bio- and Chemical Systems Technology, Reactor Engineering and Safety, Department of Chemical Engineering, KU Leuven (University of Leuven), Leuven Chem&Tech, Celestijnenlaan 200F (bus 2424), 3001 Leuven, Belgium; 4 Dentistry, University Hospitals Leuven, Kapucijnenvoer 33, 3000 Leuven, Belgium

## Abstract

Dysbiosis in the periodontal microbiota is associated with the development of periodontal diseases. Little is known about the initiation of dysbiosis. It was hypothesized that some commensal bacteria suppress the outgrowth of pathobionts by H_2_O_2_ production. However, serum and blood components released due to inflammation can neutralize this suppressive effect, leading to the initiation of dysbiosis. Agar plate, dual-species and multi-species ecology experiments showed that H_2_O_2_ production by commensal bacteria decreases pathobiont growth and colonization. Peroxidase and blood components neutralize this inhibitory effect primarily by an exogenous peroxidase activity without stimulating growth and biofilm formation of pathobionts directly. In multi-species environments, neutralization of H_2_O_2_ resulted in 2 to 3 log increases in pathobionts, a hallmark for dysbiosis. Our data show that in oral biofilms, commensal species suppress the amounts of pathobionts by H_2_O_2_ production. Inflammation can neutralize this effect and thereby initiates dysbiosis by allowing the outgrowth of pathobionts.

Although it is well known that complex poly-microbial oral biofilms are a necessity to develop tooth decay or periodontal diseases, they are often in balance with the host and consequently do not result in pathology^1^,^2^. Not their presence as such but changes in their composition and/or metabolic activity drive pathology. The current etiological model of periodontal disease, termed ‘polymicrobial synergy and dysbiosis’, proposes that changes in the periodontal microbiota or dysbiosis deregulate the host immune response, leading to chronic inflammation^3^,^4^. Although dysbiosis is characterized by a proportional increase of pathogenic species and a decrease of commensal species, little is known about the initiation of dysbiosis^4^.

Most likely, dysbiosis is initiated by a complex interplay between the bacterial community and the host. Commensal bacteria act with the host to prevent colonization or outgrowth of pathobionts that induce inflammation and disrupt the microbial ecology^5^. Pathobionts are natural members of the human microbiota that under certain perturbations to the host and/or microbiota can cause pathology^6^,^7^. Subgingival inter-bacterial correlations and competition between commensal bacteria and pathobionts in relation to disease severity have been shown^8^. As part of the oral commensal microbiota, highly abundant streptococci produce antimicrobial substances that limit the growth of pathobionts^9^. It has been shown clinically that in patients with periodontal disease, there is a decline in commensal streptococci such as *Streptococcus sanguinis*^8^,^10^. It was suggested that H_2_O_2_ is one of the most important antimicrobials produced by certain streptococci^11^. For instance, *S. sanguinis* and *Streptococcus gordonii* can inhibit the growth of *Streptococcus mutans* and *Aggregatibacter actinomycetemcomitans* by H_2_O_2_ production^12^,^13^. Recently, Rodriguez Herrero and coworkers showed that *S. oralis*, *S. gordonii*, *S. cristatus*, *S. parasanguinis*, *S. mitis* and *S. sanguinis* are good H_2_O_2_ producers, even under anaerobic conditions^11^. Although, it has been suggested that H_2_O_2_ has an important role in the formation and the composition of the oral biofilms^14^, the effect of H_2_O_2_ production on oral multi-species biofilm composition has not been investigated yet.

Gingival crevicular fluid (GFC) is an inflammatory exudate of serum that that covers oral biofilms^15^. It can modulate the composition of oral biofilms by inhibiting or enhancing the growth of certain oral species or by promoting biofilm formation and inhibiting the adhesion of certain species^16^. Additionally, serum and blood components can have peroxidase activity^17^. This is of significance since these could interact with the H_2_O_2_ produced by commensal bacteria to suppress the outgrowth of pathobionts. Clinically it is known that absence of bleeding on probing is a good marker for periodontal stability^18^. Recently, it is shown that crevicular myeloperoxidase concentrations are highly correlated with periodontal disease severity^19^.

Therefore, it can be hypothesized that commensal bacteria suppress the overgrowth of pathobionts by H_2_O_2_ but some serum and blood components released during inflammation can neutralize this suppressive effect, leading to the initiation of dysbiosis.

The objective of this study is to determine the neutralizing effect of serum, hemoglobin and hemin on the inhibitory effect of the commensal bacteria towards pathobionts.

## Results

### Decrease of the inhibitory effect of commensals on agar plates

**Figure 1:**
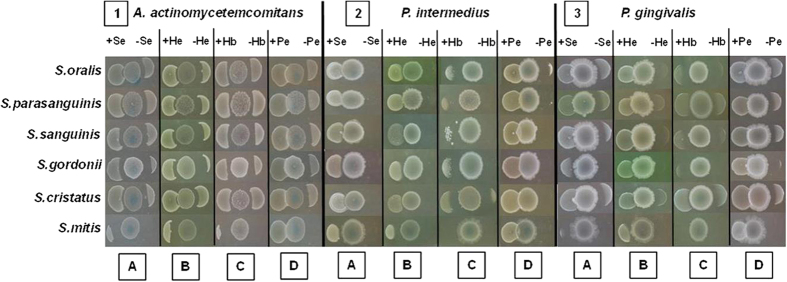
Neutralization effect of serum, hemin, hemoglobin and peroxidase on the inhibitory effect of commensal species towards *A. actinomycetemcomitans. P. intermedia* and *P. gingivalis*. The commensals were spotted 24 hours before the pathogens in the center of the pictures. The pathogens were spotted at both sides of the commensals, at the left side plus the blood compound (serum (+Se), hemin (+He), hemoglobin (+Hb) and peroxidase (+Pe)) and at the right side without any blood compound.

In order to verify the inhibitory effect of some commensal species on pathobionts and the influence of hemin, serum, hemoglobin and peroxidase, a qualitative agar-plate method was used. On agar plates, hemin, serum, hemoglobin and peroxidase significantly lowered the inhibitory effect of commensal bacteria on *P. intermedia*, *P. gingivalis* and *A. actinomycetemcomitans* (Fig. 1). This effect was observed for all commensal species, all substrates on all pathobionts with exception for the effect of hemoglobin on the inhibition of *P. intermedia* and *P. gingivalis* by *S. mitis* (Table 1). Only peroxidase was able to completely neutralize the inhibitory effect of all the commensals. In most cases, hemin lowered the inhibition of the commensals more than serum and hemoglobin.

### Decrease of the inhibitory effect of commensals in dual-species planktonic cultures and biofilms

**Figure 2:**
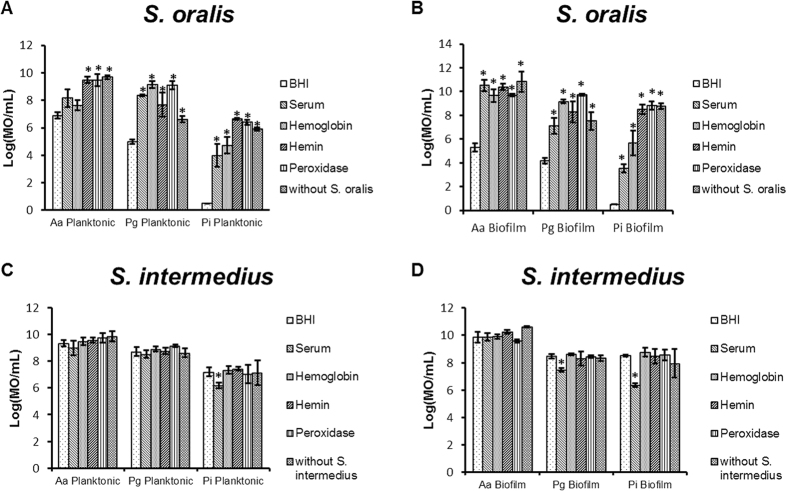
Neutralizing effect of serum and blood compounds in dual species interactions analyzed by PMA-qPCR (mean ± standard deviation, n = 3). (**A**,**B**) Represent the competition between *S. oralis* and *A. actinomycetemcomitans* (Aa), *P. gingivalis* (Pg) and *P. intermedia* (Pi) in presence of BHI, serum, hemoglobin, hemin and peroxidase in planktonic and biofilm conditions. (**C**,**D**) represents the competition between *S. intermedius* and *A. actinomycetemcomitans* (Aa), *P. gingivalis* (Pg) and *P. intermedia* (Pi) in presence of BHI, serum, hemoglobin, hemin, peroxidase in planktonic and biofilm conditions. Date are expressed as number of micro-organisms (MO)/mL. *Designates a statistically significant increase of the bacterial concentration in respect to BHI (p < 0.05).

Since bacteria within the oral cavity primarily live as biofilms, which can change their behavior, the decreased inhibitory effect induced by hemin, serum, hemoglobin and peroxidase in agar-plate experiments was verified in dual-species planktonic and biofilm cultures containing a commensal species and a pathobiont. In dual-species planktonic cultures and biofilms, the inhibitory effect of *S. oralis* on *P. intermedia*, *P. gingivalis* and *A. actinomycetemcomitans* was decreased (p < 0.05) by serum, hemin, hemoglobin and peroxidase (Fig. 2A and B). The effect was not significant for serum and hemoglobin on planktonic *A. actinomycetemcomitans*. For most of the dual-species experiments, adding serum, hemin, hemoglobin and peroxidase completely abolished the inhibitory effect of *S. oralis* resulting in numbers of pathobionts similar to the negative control in which *S. oralis* was not present.

*S. intermedius*, a non-H_2_O_2_ producing species, did not show an inhibitory effect on the pathobionts (Fig. 2C and D). Consequently serum, hemin, hemoglobin and peroxidase could not decrease an inhibitory effect on the pathobionts but they also did not increase planktonic growth and biofilm formation in dual-species experiments. However, in these experiments, serum decreased the planktonic growth of *P. intermedia* and dual species biofilm formation of *P. intermedia* and *P. gingivalis* (p < 0.05).

### Effect of serum, hemoglobin, hemin and peroxidase on single species cultures

**Figure 3:**
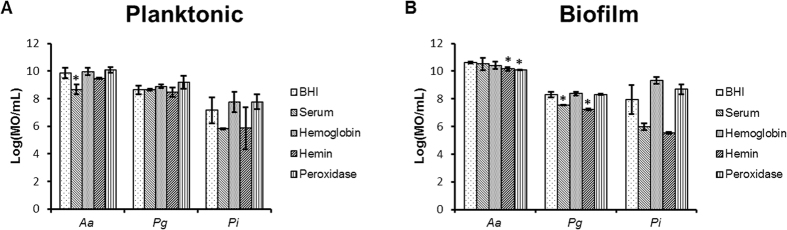
Effect of serum, hemoglobin, hemin and peroxidase on the growth (**A**) and biofilm formation (**B**) of *A. actinomycetemcomitans (Aa*), *P. gingivalis (Pg*) and *P. intermedia (Pi*) analyzed by PMA-qPCR (mean ± standard deviation, n = 3). Date are expressed as MO/mL. *Designates a statistically significant difference of the bacterial concentration in respect to BHI (p < 0.05).

In order to verify if the outgrowth of the pathobionts in the dual-species experiments was due to a decreased inhibitory effect of the commensal species and not due to an increased growth or biofilm formation of the pathobionts by the presence of hemin, serum, hemoglobin or peroxidase. The effect of these substrates on pathobiont growth and biofilm formation was evaluated. Serum, hemin, hemoglobin and peroxidase did not increase growth or biofilm formation of *P. intermedia*, *P. gingivalis* and *A. actinomycetemcomitans* (Fig. 3). Moreover, serum and hemin induced a small, but significant reduction on *P. gingivalis* biofilm formation (p < 0.05). Additionally, *A. actinomycetemcomitans* growth was decreased by serum and its biofilm formation by hemin and peroxidase (p < 0.05).

**Figure 4:**
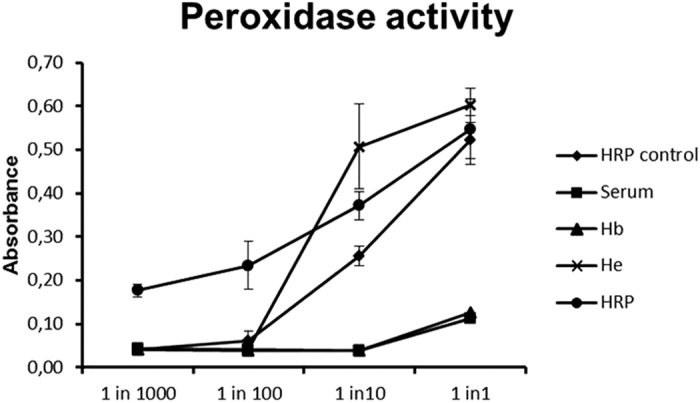
Peroxidase activity of serum and blood compounds (mean ± standard deviation, n = 3). Peroxidase activity of different dilutions of serum and blood compounds was measured via the Amplex® Red Hydrogen Peroxide/Peroxidase Assay Kit. Data are expressed as absorbance measured at 560 nm.

Since it was technically impossible to directly measure the H_2_O_2_ concentration in cultures in the presence of hemin, hemoglobin or serum with the Amplex® Red Hydrogen Peroxide kit, the peroxidase activity of hemin, serum, hemoglobin and peroxidase was determined. Although all compounds showed peroxidase activity, Peroxidase and hemin showed a higher peroxidase activity when compared to hemoglobin and serum (Fig. 4). The peroxidase activity was only observed in the highest concentrations of serum and hemoglobin whereas a peroxidase activity was also detected in diluted concentrations of hemin and peroxidase.

### Reduction of the inhibitory effect of commensals in multi-species ecologies

**Figure 5:**
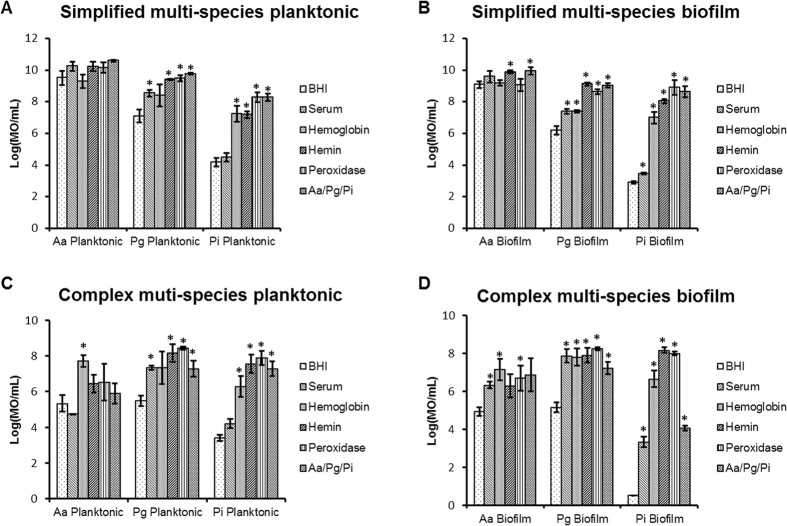
Neutralization effect of serum, hemoglobin, hemin and peroxidase on simple (3 pathobionts) and complex biofilms (14 species community) analyzed by PMA-qPCR (mean ± standard deviation, n = 3). (**A**,**B**) Represent the concentration of *A. actinomycetemcomitans* (Aa), *P. gingivalis* (Pg) and *P. intermedia* (Pi) in presence of BHI, serum, hemoglobin, hemin and peroxidase in planktonic and biofilm conditions. (**C**,**D**) represent the concentration of *A. actinomycetemcomitans* (Aa), *P. gingivalis* (Pg) and *P. intermedia* (Pi) in presence of BHI, serum, hemoglobin, hemin and peroxidase in planktonic and biofilm conditions. Date are expressed as MO/mL. *Designates a statistically significant difference of the bacterial concentration in respect to BHI (p < 0.05).

Bacteria within the oral cavity are part of complex microbial ecologies. Since the observed effects in dual-species experiments might be different in more complex ecologies, the effect of hemin, hemoglobin, serum and peroxidase on simplified and complex multi-species ecologies was examined. In both biofilm models, commensal biofilms containing *S. oralis*, *S. gordonii*, *S. cristatus*, *S. parasanguinis*, *S. mitis* and *S. sanguinis* were challenged with either only 3 pathobionts (*simplified ecology*) or with a complex 14 species ecology (*complex ecology*). The commensal biofilm significantly inhibited the planktonic and biofilm concentrations of *P. gingivalis* and *P. intermedia* both in simplified (Fig. 5A and B) and complex multi-species ecologies (Fig. 5C and D). Its effect on *A. actinomycetemcomitans* concentrations was limited in simplified multi-species ecologies (Fig. 5A and B). Although it was more pronounced in complex multi-species ecologies, it did not reach statistical significance (Fig. 5C and D). In general, serum, hemin, hemoglobin and peroxidase decreased the inhibitory effect of the commensal biofilm on planktonic and biofilm concentrations of the pathobionts. This inhibition resulted in an outgrowth of *A. actinomycetemcomitans, P. gingivalis* and *P. intermedia* respectively of up to 2.37 (±0.98), 4.48 (±0.62) and 2.94 (±0.38) log_10 _CFU/ml in complex planktonic multi-species ecologies and of up to 2.21 (±0.46), 8.17 (±0.14) and 3.12 (±0.18) log_10 _CFU/ml in complex multi-species biofilms. The neutralizing effect of serum and hemoglobin was less than that of hemin and peroxidase.

The presence of the commensal biofilm did not only result in decreased planktonic concentrations of *P. gingivalis* and *P. intermedia* (Table 2). In the planktonic ecology, also a decreased concentration of *S. salivarius* and increased concentrations of *S. sanguinis* and *S. oralis* were observed when the commensal biofilm was present (p < 0.05). On the other hand, the biofilm concentrations of *S. mutans, S. sobrinus* and *S. salivarius* were decreased and the biofilm concentrations of *F. nucleatum* and *S. sanguinis* were increased (p < 0.05).

Moreover, in the complex multi-species ecologies (Table 2), the presence of serum increased the concentration of planktonic *P. gingivalis* (p < 0.05) and the concentrations of *P. intermedia*, *P. gingivalis*, *A. actinomycetemcomitans* and *S. oralis* in the biofilm (p < 0.05). Additionally, it decreased the planktonic concentrations of *F. nucleatum*, *A. viscosus*, *S. oralis*, *S. sobrinus* and *S. gordonii* (p < 0.05) and the concentrations of *F. nucleatum*, *A. viscosus* and *A. naeslundii* in the biofilm (p < 0.05).

In contrast, the planktonic concentrations of *A. actinomycetemcomitans* and *P. intermedia* as well as the biofilm concentrations of *A. actinomycetemcomitans*, *P. gingivalis, P. intermedia, A. viscosus* and *S. sanguinis* were increased in the presence of hemoglobin (p < 0.05).

The addition of hemin increased the planktonic concentrations of *P. gingivalis* and *P. intermedia* but decreased the concentrations of *A. viscosus*, *S. mutans*, *S. gordonii* and *S. mitis* (p < 0.05). In the biofilms, hemin increased the concentrations of *P. gingivalis* and *P. intermedia* and decreased the concentrations of *A. viscosus*, *A. naeslundii*, *S. sanguinis* and *S. gordonii* (p < 0.05).

Peroxidase increased the planktonic concentrations of *P. gingivalis* and *P. intermedia* (p < 0.05) and the biofilm concentrations of *P. intermedia*, *P. gingivalis*, *A. actinomycetemcomitans* (p < 0.05).

## Discussion

Dysbiosis in oral bacterial communities is characterized by a microbial shift, which is translated in an increase of pathobionts and a decrease of commensal species^8^,^10^,^20^. It has been shown that some commensal species can suppress the growth of pathobionts by H_2_O_2_ under specific environmental conditions. More specifically, the sequence of colonization and the presence of oxygen are major influencing factors^11^. Additionally, dysbiotic biofilms are enriched in virulence factors that stimulate the host inflammatory response^21^. Although it can be deduced that both absence of commensal species and the presence inflammation are key factors, the factors driving dysbiosis are unclear^4^. Moreover, as far as the authors know, dysbiosis has never been induced in *in vitro* multi-species biofilms. In this study, it was hypothesized that commensal bacteria suppress the overgrowth of pathobionts by H_2_O_2_ but some serum and blood components released during inflammation can neutralize this suppressive effect, leading dysbiosis. It was shown on agar plates and in dual-species biofilms that H_2_O_2_ production by commensal bacteria decreases pathobiont growth and colonization. Although it was not directly tested in the current study, Herrero *et al.* showed that the amount of inhibition on pathobiont growth is determined by oxygen availability^11^. Commensal bacteria could produce H_2_O_2_ and inhibit pathobiont growth under anaerobic condition. However, H_2_O_2_ production and pathobiont inhibition was significantly higher under aerobic conditions. Therefore, oxygen availability must play an important role into the transition from homeostasis to dysbiosis^11^. If a non-H_2_O_2_ producing species was used in the current study, no inhibition was observed. Peroxidase and blood components neutralize the inhibitory effect of H_2_O_2_ primarily by a peroxidase activity since they did not stimulate the growth and biofilm formation of the pathobionts directly. In multi-species environments, neutralization of H_2_O_2_ by peroxidase or blood components resulted in 2 to 3 log increases in pathobionts which can be considered as a hallmark for dysbiosis.

The agar plate experiments showed an inhibition of the pathobionts when grown in the vicinity of the commensals. This inhibition was completely neutralized in the presence of peroxidase. These results were in concordance with previous studies^11^,^12^,^22^ that also identified H_2_O_2_ as the main inhibitory substance. It was observed that the magnitudes of inhibition described by Van Essche *et al.* were markedly smaller than the ones reported in the current study although the same bacterial strains were used^23^. This was attributed to the blood agar medium which was used in the Van Essche study and pointed towards an interference of certain blood compounds on the inhibition effect of these streptococcus species.

Gingival inflammation is characterized by an increase in GCF production, which is similar to serum, and bleeding tendency. Among many other components, blood contains serum, hemin and hemoglobin^24^ but the hemolytic capacity of pathobionts can also increase their concentration^25^. It is already described that hemoglobin and hemin have a peroxidase activity^17^,^26^,^27^. *P. intermedia* and *P. gingivalis* also generate layers of haems with catalytic activity to degrade the H_2_O_2_ using hemin and hemoglobin^28^. Additionally, serum and GCF contains catalytic molecules such as myeloperoxidase that neutralizes the antimicrobial effect of H_2_O_2_^19^. Recently, it is shown that crevicular myeloperoxidase concentrations are highly correlated with periodontal disease severity. All these studies substantiate the observation that hemin, hemoglobin and serum decrease the inhibitory effect of the commensal species on agar plates by H_2_O_2_ reduction. The effects of hemin, hemoglobin, serum and the role of H_2_O_2_ were further demonstrated in dual- and multi-species biofilms. Their addition resulted in increases in pathobionts of up to 8 log values both in planktonic and in biofilm cultures. Although, it has been suggested that hemin and hemoglobin can increase the growth and biofilm formation of some pathogens, this effect was not seen^29^,^30^,^31^,^32^ (Fig. 3). This can be explained by the qPCR method used to quantify growth and biofilm formation and the growth medium used in this study which might provide sufficient iron sources to the pathobionts so that addition of hemin and hemoglobin did not affected their growth or biofilm formation anymore. The effect of serum on pathobiont growth and biofilm formation is even less reported in literature. Biyikoĝlu *et al.* reported no effects of serum on *A. odontolyticus*, *V. parvula*, *F. nucleatum* and *P. gingivalis* over time but a decrease in *F. nucleatum* biovolume when 4 species biofilms were grown in the presence of serum^16^. These data are in accordance with the present data.

**Figure 6:**
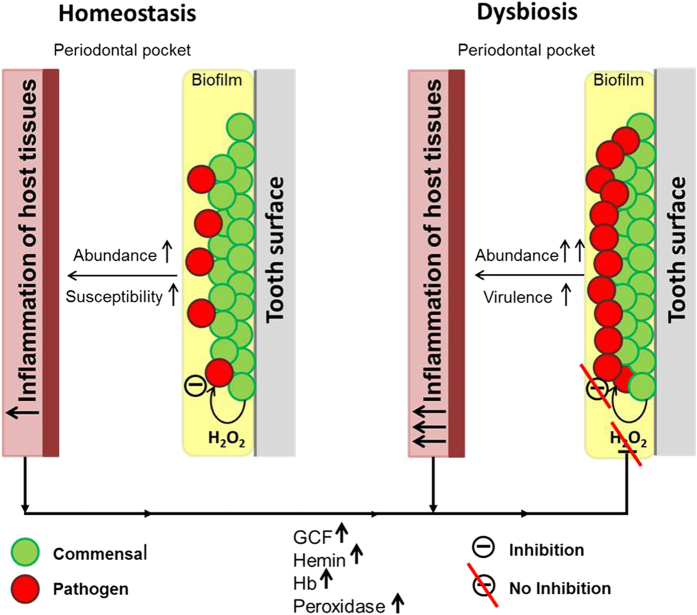
Hypothetical model on the H_2_O_2_ neutralizing effect of hemin, hemoglobin and peroxidase on the inhibitory effect of commensal species towards periodontal pathobionts during inflammation. The neutralization of H_2_O_2_ results in the outgrowth of periodontal pathobionts, leading to the initiation of dysbiosis in oral biofilms.

Overall, the study suggests that serum and blood compounds play an important role in the initiation of dysbiosis of oral biofilms by disrupting the inhibitory defensive barrier provided by the commensal bacteria. The data provide a hypothesis for the initiation of dysbiosis in oral biofilms (Fig. 6). In oral biofilms some commensal species can suppress the amounts of pathobionts by H_2_O_2_ generation. However, when these biofilms persist over longer periods of time or become more abundant or when the susceptibility of the host changes, the resulting inflammatory reaction neutralizes the effects of H_2_O_2_, thereby allowing the outgrowth of pathobionts. Additionally, changes in the oxygen availability within the biofilm might also lower the H_2_O_2_ production and subsequently contribute to the outgrowth of pathobionts.

## Methods

### Bacterial strains and media

All used bacterial species (*Streptococcus sanguinis* LM14657, *Streptococcus cristatus* ATCC 49999, *Streptococcus gordonii* ATCC 49818, *Streptococcus parasanguinis* DSM 6778, *Streptoccocus mitis* DSM 12643, *Streptoccocus oralis* DSM 20627, *Streptococcus salivarius* TOVE-R, *Streptococcus intermedius DSM 20573, Streptococcus mutans ATCC 20523, Streptococcus sobrinus ATCC 20742, Actinomyces viscosus* DSM 43327, *Actinomyces naeslundii* ATCC 51655, *Prevotella intermedia* ATCC 25611, *Porphyromonas gingivalis* ATCC 33277, *Fusobacterium nucleatum* ATCC 20482, *Aggregatibacter actinomycetemcomitans* ATCC 43718 and *Veillonella parvula* DSM 2008) were maintained on blood agar (Oxoid, Basingstoke, UK) supplemented with 5 mg/mL hemin (Sigma, St. Louis, USA), 1 mg/mL menadione (Calbiochem-Novabiochem, La Jolla, USA) and 5% sterile horse blood (E&O Laboratories, Bonnybridge, Scotland). Overnight liquid cultures were prepared in Brain Hearth Infusion (BHI) broth (Difco, Detroit, USA). Competitive inhibition experiments were performed in Brain Hearth Infusion 2 (BHI-2) broth or agar containing Brain Heart infusion (Difco, Detroit, USA) supplemented with 2.5 g/L mucin (Sigma-Aldrich, St. Louis, USA), 1.0 g/L yeast extract (Oxoid, Basingstoke, UK), 0.1 g/L cysteine (Calbiochem, San Diego, USA), 2.0 g/L sodium bicarbonate and 0.25% (v/v) glutamic acid (Sigma-Aldrich, St Louis, USA). The bacteria were cultured under aerobic (5% CO_2_) or anaerobic (80% N_2_, 10% H_2_ and 10% CO_2_) conditions. Optical densities were measured and adjusted using spectrophotometry (OD600, GeneQuant Spectrophotometer, Buckinghamshire, UK).

### Serum and blood components

Hemin, human hemoglobin and horseradish peroxidase (Sigma-Aldrich, St. Louis, USA) were dissolved in BHI-2 at concentrations of 5 mg/mL hemin, 0.44 mg/mL hemoglobin^33^ and 16 μg/mL peroxidase. Human serum was obtained by venipuncture of a single, systemically healthy, male volunteer with no oral disease and who had not taken any antibiotics for 1 year. Peripheral venous blood was immediately centrifuged at 264 × g for 30 min at room temperature. The serum was removed and frozen at −20 °C after aliquotation.

### Ethics Statement

The use of human serum was approved by the ethical committee of the KU Leuven and registered with identifier B322201628215. The procedures were executed according to the Helsinki Declaration and the regulations of the University Hospital, which are approved by the ethical committee. The adult subject provided a written and oral consent after having explained to him the purpose of the study. The subject is aware that the results will be used in a scientific study. An informed consent was obtained from all subjects.

### Antagonistic experiments on agar plates

The spotting technique was used to quantify the inhibitory effect of 6 commensal species on 3 pathobionts and to identify neutralization effects by serum and blood components^11^. An overnight culture of a commensal species was adjusted to a concentration of 10^9^ CFU/mL. This solution was spotted on an agar plate and incubated under aerobic conditions. After 24 hours, an overnight culture of the pathobiont (10^9^ CFU/mL) was spotted next to the commensal spot. After 48 hours of anaerobic incubation, a calibrated photograph was taken from the agar plate and the magnitude of inhibition was measured from the edge of the commensal colony to the border of the inhibited pathobiont colony using ImageJ (http://rsb.info.nih.gov/ij/download.html).

### Dual-species planktonic and biofilm experiments

*S. oralis* was selected as the model commensal species with a strong inhibitory effect on pathobionts by H_2_O_2_ production. *S. intermedius* was used as a not-inhibiting commensal species (negative control)^11^. 10 mL of an overnight culture of these commensals was centrifuged (1438 × g, 10 minutes). The supernatant was discarded and the pellet was re-suspended in 10 mL BHI-2 broth. The density was adjusted to 1 × 10^8^ CFU/ml. 6 ml of this solution was transferred to 6 wells (1 ml/well) of a 24 well-plate (Greiner, Frickenhausen, Germany) and incubated under aerobic conditions. After 24 hours, 500 μL of BHI-2 broth, serum, hemoglobin, hemin or peroxidase was added to the cultures. Additionally, an overnight culture of a pathobiont (*A. actinomycetemcomitans, P. gingivalis* or *P. intermedia*) was centrifuged (1438 × g, 10 minutes) and re-suspended in BHI-2 (1 × 10^8^ CFU/ml). 1 mL of this bacterial solution was inoculated in each well with *S. oralis* or *S. intermedius* and in an additional well containing 1.5 mL BHI-2 (negative control). After 24 hours of anaerobic incubation, 1 mL was taken from each well, centrifuged (1438 × g, 10 min), re-suspended in PBS and analyzed via vitality q-PCR. Afterwards, the remaining supernatant was removed and the biofilms at the bottom of the wells were washed with phosphate buffered saline (PBS). The biofilms were detached with 500 μL 0,05% Trypsin-EDTA (Gibco, Paisley, UK) for 15 minutes at 37 °C, transferred to Eppendorf tubes, centrifuged (6010 × g, 10 minutes) and after discarding the trypsin, the biofilm pellets were re-suspended in 1 mL of PBS and analyzed by vitality q-PCR.

### Effects on the growth and biofilm formation of pathobionts

An overnight culture of a pathobiont (*A. actinomycetemcomitans, P. gingivalis* or *P. intermedia*) was centrifuged (1438 × g, 10 minutes) and re-suspended in BHI-2 (1 × 10^8^ CFU/mL). 1 mL of this bacterial solution was inoculated in each well plus 500 μl of BHI-2 broth, serum, hemoglobin, hemin or peroxidase. The wells were incubated for 24 hours under anaerobic conditions where after planktonic bacteria and the biofilms analyzed as described above.

### Peroxidase activity of blood compounds and peroxidase on H_2_O_2_

50 μl of a 100 μM H_2_O_2_ solution was mixed with either 10 μl horseradish peroxidase (10 U/ml) (control) or 10 μl serum or 10 μl hemoglobin (0.44 mg/ml) or 10 μl hemin (5 mg/ml) or 10 μl peroxidase (16 μg/ml). Additionally, 50 μl of a 100 μM H_2_O_2_ solution was mixed with 10 fold serial dilutions of peroxidase, serum, hemoglobin, hemin and horseradish peroxidase in PBS (up to 1 in 1000). To 50 μl of these solutions, 50 μl of Amplex Red (50 μM) was added (Amplex® Red Hydrogen Peroxide/Peroxidase Assay Kit, Life technologies). After 20 minutes, the absorbance was measured at 560 nm (Powerwave XS Microplate Spectrophotometer, BioTek Instruments, Winooski, USA) according to the manufacturer’s instructions in order to determine the peroxidase activity of serum, hemin, hemoglobin and peroxidase.

### Simplified multi-species planktonic and biofilm experiments

Similar to the dual-species experiments, overnight cultures of six commensal bacteria (*S. oralis*, *S. gordonii*, *S. cristatus*, *S. parasanguinis*, *S. mitis* and *S. sanguinis*), with inhibitory effects by producing H_2_O_2_, were centrifuged, re-suspended in BHI-2 broth (1 × 10^8^ CFU/mL). Equal volumes of these solutions were mixed and inoculated in 6 wells of a 24 well-plate and incubated under aerobic conditions. After 24 hours, BHI-2, serum, hemoglobin, hemin and peroxidase were added to the wells as described above. Additionally, 1 mL of an overnight co-culture of 3 pathobionts (*A. actinomycetemcomitans, P. gingivalis* and *P. intermedia*) was centrifuged (1438 × g, 10 minutes), re-suspended in BHI-2 (1 × 10^8^ CFU/ml) and added to the wells. The latter co-culture was obtained from overnight cultures of the pathobionts (*simplified ecology*) which were centrifuged (1438 × g, 10 minutes) and re-suspended in 10 ml of BHI-2. 1 mL of each pathobiont culture was added to 7 mL of BHI-2 and incubated for 24 hours under anaerobic conditions to obtain the co-culture. The wells were incubated for 24 hours under anaerobic conditions where after planktonic bacteria and the biofilms analyzed as described above.

### Complex multi-species planktonic and biofilm experiments

The experimental set-up was identical to the set-up used for the simplified multi-species experiments with the exception that instead of using an overnight co-culture of 3 pathobionts, a bioreactor derived complex multi-species co-culture of 14 species (*complex ecology*), as described below, was used. The wells were incubated for 24 hours under anaerobic conditions where after planktonic bacteria and the biofilms analyzed as described above.

### Bioreactor derived multi-species community

A multi-species community was established in a BIOSTAT B TWIN (Sartorius, Germany) bioreactor. 750 mL of BHI-2 broth was added to the vessel together with 5.0 mg/mL hemin, 1.0 mg/mL menadione and 200 μl/L Antifoam Y-30 (Sigma, St. Louis, USA). The medium was pre-reduced over 24 hours at 37 °C by bubbling 100% N_2_ and 5% CO_2_ in the medium under continuous stirring at 300 rpm. pH was set at 6.7 +/−0.1. After 24 hours, overnight cultures of *S. sanguinis*, *S. gordonii*, *S. salivarius*, *S. mitis*, *S. oralis*, *S. mutans*, *S. sobrinus*, *A. viscosus*, *A. naeslundii*, *P. intermedia*, *P. gingivalis*, *F. nucleatum*, *A. actinomycetemcomitans* and *V. parvula* were adjusted to an OD of 1.4 and added to the bioreactor. During the first 48 hours, the medium was not replaced. After that, the medium was replaced at a rate of 200 mL/24 hours.

### Vitality q-PCR

DNA extraction and vitality q-PCR using propidium monoazide was previously described^34^. Table 3 shows primer and probe sequences used in this study.

### Statistical analysis

All experiments were repeated on 3 different days. To account for the censored character of the inhibition data from agar plates experiments, differences between treatments (with serum, hemoglobin, hemin or peroxidase) and control (without addition of blood compound or peroxidase) for the inhibition data were analyzed by means of a survival regression model for gaussian data. Comparisons between treatments and control were made for each combination of substance (blood compounds or peroxidase) and bacteria and corrected for simultaneous hypothesis testing according to Sidak. For planktonic and biofilm data, a linear mixed model was fit to model the log-transformed CFU counts using substrate (blood compounds or peroxidase) and sample type (planktonic or biofilm) as fixed factors and run as random factor. Since a residual analysis showed that the model was heteroscedastic, weights, proportional to the inverse of the predicted value, were applied. Comparisons with BHI and control were made separately by calculating the appropriate contrasts and a correction for simultaneous hypothesis testing according to Dunnett was applied. Data were analyzed using S-plus 8.0 for Linux (Tibco, Palo Alto, CA, USA).

## Additional Information

**How to cite this article**: Herrero, E. R. *et al.* Dysbiosis by neutralizing commensal mediated inhibition of pathobionts. *Sci. Rep.* **6**, 38179; doi: 10.1038/srep38179 (2016).

**Publisher's note:** Springer Nature remains neutral with regard to jurisdictional claims in published maps and institutional affiliations.

## Figures and Tables

**Table 1:** Effect of the addition of serum, hemoglobin, hemin and peroxidase to BHI-2 agar on the antagonistic activity of commensal species.

		Serum		Hemoglobin		Hemin		Peroxidase	
		−	+	−	+	−	+	−	+
*S. oralis*	Aa	2,04 ± 0,07	0,62 ± 0,17*	2,08 ± 0,27	1,33 ± 0,21*	2,35 ± 0,23	0,67 ± 0,03*	2,31 ± 0,38	0,00 ± 0,00*
	Pi	TI	1,43 ± 0,08*	TI	2,71 ± 0,25*	3,63 ± 0,81	0,46 ± 0,20*	TI	0,00 ± 0,00*
	Pg	TI	1,37 ± 0,11*	4,36 ± 0,37	2,58 ± 0,24*	2,23 ± 0,02	0,00 ± 0,00*	2,67 ± 0,16	0,00 ± 0,00*
*S. sanguinis*	Aa	3,04 ± 0,04	1,45 ± 0,17*	2,67 ± 0,42	1,94 ± 0,21*	2,70 ± 0,25	1,14 ± 0,03*	3,08 ± 0,57	0,00 ± 0,00*
	Pi	TI	1,29 ± 0,07*	3,37 ± 0,14	1,41 ± 0,28*	2,75 ± 0,32	0,25 ± 0,19*	TI	0,00 ± 0,00*
	Pg	4,58 ± 0,11	0,78 ± 0,15*	3,04 ± 0,12	2,17 ± 0,05*	2,63 ± 0,39	0,00 ± 0,00*	3,65 ± ± 0,35	0,00 ± 0,00*
*S. parasanguinis*	Aa	1,99 ± 0,04	0,75 ± 0,03*	1,41 ± 0,27	0,47 ± 0,11*	2,52 ± 0,14	0,80 ± 0,02*	1,80 ± 0,24	0,00 ± 0,00*
	Pi	TI	0,00 ± 0,00*	TI	3,05 ± 0,05*	TI	3,64 ± 0,10*	TI	0,00 ± 0,00*
	Pg	2,63 ± 0,24	0,00 ± 0,00*	1,14 ± 0,07	0,71 ± 0,10	1,62 ± 0,12	0,00 ± 0,00*	1,87 ± 0,27	0,00 ± 0,00*
*S. mitis*	Aa	TI	2,83 ± 0,09*	5,26 ± 0,85	2,56 ± 0,23*	TI	2,37 ± 0,05*	TI	0,00 ± 0,00*
	Pi	TI	1,76 ± 0,05*	TI	TI	TI	1,36 ± 0,29*	TI	0,00 ± 0,00*
	Pg	TI	2,58 ± 0,16*	TI	TI	TI	1,29 ± 0,13*	TI	0,00 ± 0,00*
*S. gordonii*	Aa	2,68 ± 0,04	1,05 ± 0,11*	3,07 ± 0,21	2,41 ± 0,26*	3,75 ± 0,07	1,25 ± 0,02*	2,11 ± 0,29	0,00 ± 0,00*
	Pi	TI	1,20 ± 0,13*	TI	2,78 ± 0,08*	4,42 ± 0,52	0,78 ± 0,31*	TI	0,00 ± 0,00*
	Pg	TI	0,24 ± 0,17*	TI	3,40 ± 0,15*	3,55 ± 1,02	0,31 ± 0,08*	3,21 ± 0,44	0,00 ± 0,00*
*S. cristatus*	Aa	2,25 ± 0,15	0,94 ± 0,09*	2,00 ± 0,32	1,16 ± 0,18*	1,57 ± 0,06	0,45 ± 0,08*	2,02 ± 0,33	0,00 ± 0,00*
	Pi	TI	0,82 ± 0,15*	TI	2,49 ± 0,44*	TI	1,33 ± 0,21*	TI	0,00 ± 0,00*
	Pg	2,96 ± 0,26	0,00 ± 0,00*	3,05 ± 0,22	1,98 ± 0,24*	1,61 ± 0,15	0,00 ± 0,00*	2,98 ± 0,41	0,00 ± 0,00*

Data represent the magnitude of the zone of inhibition expressed in mm (mean ± standard deviation, n = 3) against *A. actinomycetemcomitans* (Aa), *P. intermedia* (Pi) and *P. gingivalis* (Pg). *Statistically significant decrease of the inhibitory effect from commensal bacteria by serum, hemoglobin, hemin and peroxidase (p < 0.05). TI = Total Inhibition. − = without addition; + = with addition, 0,00 = no inhibition.

**Table 2:** Log (MO/ml) of the different species (mean ± standard deviation, n = 3) that form part of the 14 species community exposed to serum, hemoglobin, hemin and peroxidase.

	Planktonic						Biofilm					
	BHI	Serum	Hemoglobin	Hemin	Peroxidase	Control	BHI	Serum	Hemoglobin	Hemin	Peroxidase	Control
*Aa*	5.34 ± 0.46	4.73 ± 0.03	7.72 ± 0.34*	6.45 ± 0.53	6.55 ± 1.03	5.90 ± 0.55	4.93 ± 0.22	6.34 ± 0.19*	7.14 ± 0.56*	6.29 ± 0.60	6.69 ± 0.67*	6.88 ± 0.88
*Pi*	3.41 ± 0.17	4.21 ± 0.26	6.30 ± 0.57*	7.57 ± 0.42*	7.89 ± 0.38*	7.29 ± 0.42*	0.00 ± 0.00	3.33 ± 0.27*	6.65 ± 0.43*	8.17 ± 0.14*	7.99 ± 0.09*	4.05 ± 0.14*
*Pg*	5.49 ± 0.30	7.34 ± 0.12*	7.34 ± 0.90	8.17 ± 0.19*	8.44 ± 0.07*	7.30 ± 0.45*	5.15 ± 0.25*	7.87 ± 0.36*	7.81 ± 0.44*	7.91 ± 0.39*	8.25 ± 0.08*	7.23 ± 0.32*
*Fn*	9.23 ± 0.42	6.51 ± 0.21^#^	9.40 ± 0.47	9.36 ± 0.39	9.74 ± 0.35	9.49 ± 0.26	10.42 ± 0.13	6.68 ± 0.59^#^	10.64 ± 0.09	10.30 ± 0.04	10.04 ± 0.23	9.55 ± 0.10^#^
*A. naeslundii*	2.97 ± 0.05	2.99 ± 0.19	3.46 ± 0.64	2.87 ± 0.11	3.51 ± 0.82	4.52 ± 0.94	7.07 ± 0.26	5.01 ± 0.40^#^	6.87 ± 0.09	3.62 ± 1.54^#^	5.35 ± 0.98	7.32 ± 0.09
*A. viscosus*	6.87 ± 0,41	3.95 ± 0.52^#^	6.98 ± 0.21	3.89 ± 0.34^#^	6.74 ± 0.44	6.62 ± 0.13	8.03 ± 0.46	6.45 ± 0.74^#^	8.25 ± 0.42*	4.79 ± 1.15^#^	8.09 ± 0.27	7.81 ± 0.36
*S. mutans*	6.83 ± 0.05	5.66 ± 0.78	6.68 ± 0.11	5.75 ± 0.28^#^	5.90 ± 0.75	7.28 ± 0.18	7.40 ± 0.21	7.53 ± 0.45	7.58 ± 0.11	6.97 ± 0.10	7.27 ± 0.01	8.33 ± 0.09*
*S. sobrinus*	6.51 ± 0.26	5.44 ± 0.51^#^	6.34 ± 0.19	6.10 ± 0.28	5.71 ± 0.30^#^	7.08 ± 0.14	7.35 ± 0.27	7.17 ± 0.45	7.57 ± 0.17	7.53 ± 0.32	6.92 ± 0.05	9.01 ± 0.10*
*S. sanguinis*	6.15 ± 0.30	5.85 ± 0.06	6.10 ± 0.29	5.42 ± 0.33	6.09 ± 0.17	5.06 ± 0.18^#^	7.63 ± 0.09	7.69 ± 0.10	7.79 ± 0.10*	6.91 ± 0.26^#^	7.71 ± 0.05	6.31 ± 0.10^#^
*S. gordonii*	8.57 ± 0.30	7.43 ± 0.02^#^	8.46 ± 0.31	6.92 ± 0.20^#^	8.48 ± 0.25	9.17 ± 0.29	9.32 ± 0.24	9.35 ± 0.13	9.41 ± 0.19	7.76 ± 0.35^#^	9.32 ± 0.03	9.56 ± 0.08
*S. oralis*	7.68 ± 0.24	5.90 ± 0.11^#^	7.54 ± 0.38	7.75 ± 0.48	7.81 ± 0.24	6.81 ± 0.17^#^	8.43 ± 0,07	8.86 ± 0.10*	8,64 ± 0.06	8.09 ± 0.19	8.79 ± 0.16	8.04 ± 0.21
*S. salivarius*	3.44 ± 0.27	3.99 ± 0.40	3.41 ± 0.20	3.01 ± 0.06	3.73 ± 0.29	6.23 ± 0.17*	4.27 ± 0.28	4.42 ± 0.27	4.39 ± 0.41	4.23 ± 0.52	4.14 ± 0.68	6.41 ± 0.13*
*V. parvula*	7.60 ± 0.19	6.66 ± 0.89	7.39 ± 0.14	7.74 ± 0.18	7.08 ± 0.19	7.57 ± 0.12	10.06 ± 0.38	9.67 ± 0.65	10.62 ± 0.13	10.36 ± 0.03	10.56 ± 0.19	10.27 ± 0.12
*S. mitis*	4.09 ± 0.14	3.48 ± 0.19	3.69 ± 0.11	2.74 ± 0.06^#^	3.51 ± 0.08	2.93 ± 0.09	4.15 ± 0.22	4.34 ± 0.31	4.20 ± 0.23	3.24 ± 0.49	4.27 ± 0.20	2.65 ± 0.20

Control condition refers to a 14 species community without the addition of the 6 commensal bacteria. *Designates a statistically significant increase of the bacterial concentration in respect to BHI (p < 0.05). ^#^Designates a statistically significant decrease of the bacterial concentration in respect to BHI (p < 0.05).

**Table 3:** Primers and probes used for the detection and quantification by vitality qPCR.

STRAIN		Primer/Probe (5′-3′)	Fragment length
*Aggregatibacter actinomycetemcomitans*	Forward	GAA CCT TAC CTA CTC TTG ACA TCC GAA	80 bp
	Reverse	TGC AGC ACC TGT CTC AAA GC	
	Probe	AGA ACT CAG AGA TGG GTT TGT GCC TTA GGG	
*Fusobacterium nucleatum*	Forward	GGA TTT ATT GGG CGT AAA GC	162 bp
	Reverse	GGC ATT CCT ACA AAT ATC TAC GAA	
	Probe	CTC TAC ACT TGT AGT TCC G	
*Porphyromonas gingivalis*	Forward	GCG CTC AAC GTT CAG CC	68 bp
	Reverse	CAC GAA TTC CGC CTG C	
	Probe	CAC TGA ACT CAA GCC CGG CAG TTT CAA	
*Prevotella intermedia*	Forward	CGG TCT GTT AAG CGT GTT GTG	99 bp
	Reverse	CAC CAT GAA TTC CGC ATA CG	
	Probe	TGG CGG ACT TGA GTG CAC GC	
*Streptococcus mutans*	Forward	GCC TAC AGC TCA GAG ATG CTA TTC T	114 bp
	Reverse	GCC ATA CAC CAC TCA TGA ATT GA	
	Probe	TGG AAA TGA CGG TCG CCG TTA TGA A	
*Streptococcus sobrinus*	Forward	TTC AAA GCC AAG ACC AAG CTA GT	88 bp
	Reverse	CCA GCC TGA GAT TCA GCT TGT	
	Probe	CCT GCT CCA GCG ACA AAG GCA GC	
*Actinomyces naeslundii*	Forward	TCG AAA CTC AGC AAG TAG CCG	96 bp
	Reverse	AGA GGA GGG CCA CAA AAG AAA	
	Probe	GGG TAC TCT AGT CCA AAC TGG CGG ATA GCG	
*Streptococcus gordonii*	Forward	CGG ATG ATG CTA ATC AAG TGA CC	177 bp
	Reverse	GTT AGC TGT TGG ATT GGT TGC C	
	Probe	AGA ACA GTC CGC TGT TCA GAG CAA	
*Actinomyces viscosus*	Forward	GTG AAG GAG CCA GCT TGC TGG TTC TG	155 bp
	Reverse	CGG AAC AAA CCT TTC CCA GGC	
	Probe	ATG AGT GGC GAA CGG GTG AGT AAC	
*Streptococcus salivarius*	Forward	AAC GTT GAC CTT ACG CTA GC	192 bp
	Reverse	ACC GTA ACG TGG GAA AAC TG	
	Probe	GTA GCG TCA GAG TGG TTG AC	
*Streptococcus oralis*	Forward	ACC AGC AGA TAC GAA AGA AGC AT	229 bp
	Reverse	AGG TTC GGG CAA GCG ATC TTT CT	
	Probe	AAG GCT GCT GTT GCT GAA GAA GT	
*Streptococcus mitis*	Forward	GGC TCG TAG TCT GGA GAT GG	133 bp
	Reverse	TAG GTC GTC GTC CCA AGG AA	
	Probe	CGA AGA GCA CCA ATA GCA CCT CCC	
*Streptococcus sanguinis*	Forward	CAA AAT TGT TGC AAA TCC AAA GG	75 bp
	Reverse	GCT ATC GCT CCC TGT CTT TGA	
	Probe	AAA GAA AGA TCG CTT GCC AGA ACC GG	
*Veillonella parvula*	Forward	GAC GAA AGT CTG ACG GAG CA	171 bp
	Reverse	TGC CAC CTA CGT ATT ACC GC	
	Probe	AGC TCT GTT AAT CGG GAC GAA AGG C	
